
JOURNAL INFORMATION
==============================
NLM Title Abbreviation: ScientificWorldJournal
Journal ID: ScientificWorldJournal
NLM Journal ID: 101131163
Journal ID: TSWJ

The Scientific World Journal

ISSN: 2356-6140
EISSN: 1537-744X
Publisher: Wiley

ARTICLE INFORMATION
==============================
PMCID: PMC5823197
PMID: 20024508
DOI: 10.1100/tsw.2009.165
Article ID: 546241
Article version: 1
Subjects: Clinical Images

Horseshoe Kidney in an 80 Year Old with Chronic Kidney Disease

Ramkumar Hema
Ahmed Syed M. *
Syed E.
Tuazon J.
Department of Medicine, Division of Nephrology and Hypertension, Feinberg School of Medicine, Northwestern University Chicago, IL, USA
*Syed M. Ahmed: smus.ahmed@gmail.comAcademic Editor: Daniel Batlle

Publication date: 2009
Electronic publication date: 2009 Dec 16
Volume: 9
First page: 1346
Last page: 1347
Received 2009 May 11; Revised 2009 Dec 3; Accepted 2009 Dec 4
Copyright: Copyright © 2009 Hema Ramkumar et al.
Copyright year: 2009
License: This is an open access article distributed under the Creative Commons Attribution License, which permits unrestricted use, distribution, and reproduction in any medium, provided the original work is properly cited.
License URL: https://creativecommons.org/licenses/by/3.0/

==============================
Clinical Image
*TheScientificWorldJOURNAL* (2009) 9, 1346–1347
ISSN 1537-744X; DOI 10.1100/tsw.2009.165

TheScientificWorldJOURNAL
www.thescientificworld.com

### Horseshoe Kidney in an 80 Year Old with Chronic Kidney Disease

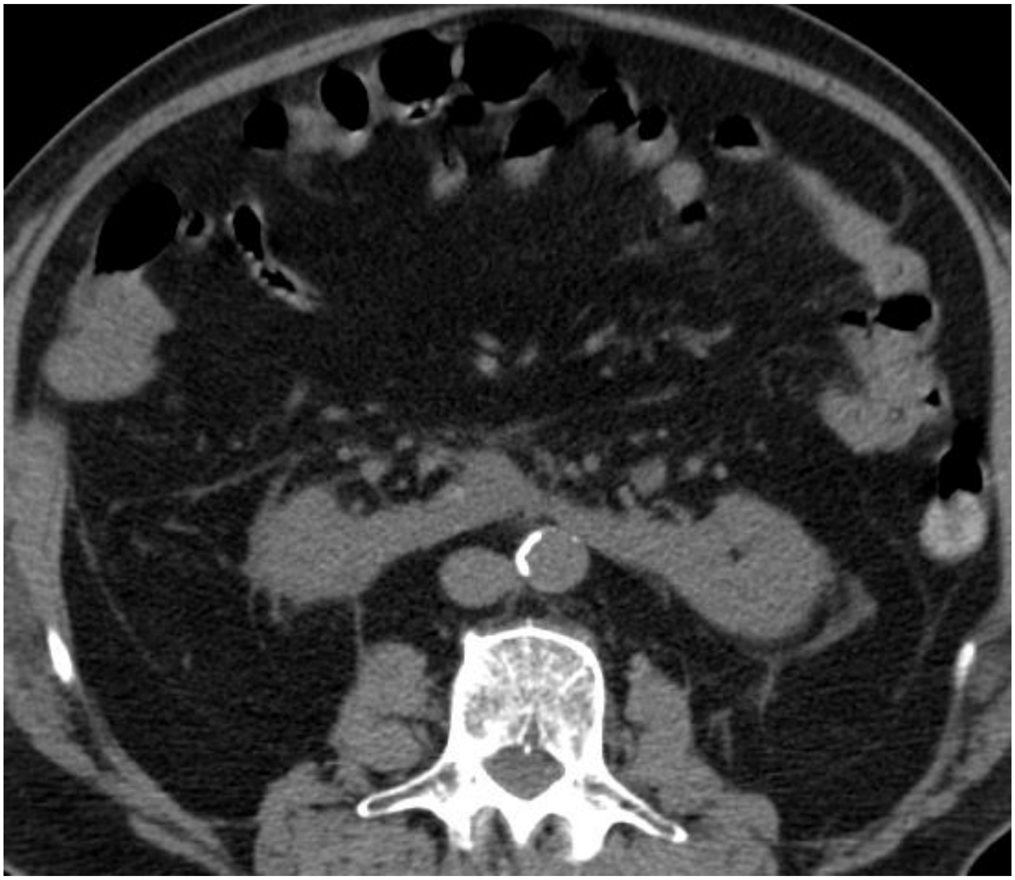

**FIGURE 1.** This transverse CT section of the abdomen shows complete fusion of the lower poles of the kidneys with a clear isthmus. The fused kidneys are sitting lower in the pelvis compared to the location of normal kidneys.

Hema Ramkumar, Syed M. Ahmed*, E. Syed, and J. Tuazon

*Department of Medicine, Division of Nephrology and Hypertension, Feinberg School of Medicine, Northwestern University Chicago, IL*

**E-mail**: smus.ahmed@gmail.com

*Received May 11, 2009; Revised December 3, 2009; Accepted December 4, 2009; Published December 16, 2009*

A horseshoe kidney is the most common renal fusion anomaly, with an incidence of 1/400 worldwide and a male-to-female ratio of 2:1. One theory of the abnormal fusion of the kidneys is that a variation in

*Corresponding author.
©2009 with author.
Published by TheScientificWorld; www.thescientificworld.com growth of pelvic structures brings the metanephric blastemas close together before renal capsule maturation, resulting in fusion. Consequently, the normal embryonic ascent of the fused kidney is disrupted by the inferior mesenteric artery, leaving the adult horseshoe kidney in a lower position than normal. About 90% of the time, the lower poles are fused and the excretory renal units and ureters are maintained separately. In the pediatric setting, 90% of patients are asymptomatic and the most common presentation is a urinary tract infection (UTI). One-third of cases are associated with other congenital or chromosomal anomalies. About one-third of all patients with a horseshoe kidney are asymptomatic. Common complications of a horseshoe kidney abnormality include uretropelvic junction obstruction, recurrent renal calculi, recurrent infections secondary to urine stasis and vesicoureteric reflux, and an increased incidence of renal tumors.

We describe the clinical image of an 80-year-old man with a history of type II diabetes, hypertension, and chronic kidney disease (CKD) who was being evaluated for abdominal pain. A computerized tomography (CT) scan (Fig. 1) of the abdomen was requested and revealed the presence of a horseshoe kidney. A horseshoe kidney does not cause progressive kidney disease. This patient, however, had CKD (estimated GFR of about 20 ml/min), most likely associated with type II diabetes and hypertension, and that was unrelated to the horseshoe kidney, which was an incidental finding.

### REFERENCES

1. Cook, W.A. and Stephens, F.D. (1977) Fused kidneys: morphologic study and theory of embryogenesis. *Birth Defects Orig. Artic. Ser.* **13(5),** 327–340.
2. Grainger, R., Murphy, D.M., and Lane, V. (1983) Horseshoe kidney--a review of the presentation, associated congenital anomalies and complications in 73 patients. *Ir. Med. J.* **76(7),** 315–317.
3. Schacht, M.J., Sakowicz, B., and Rao, M.S. (1983) Intermittent abdominal pain in a patient with horseshoe kidney. *J. Urol.* **130(4),** 749–751.
4. Boatman, D.L., Kolln, C.P., and Flocks, R.H. (1972) Congenital anomalies associated with horseshoe kidney. *J. Urol.* **107(2),** 205–207.

**This article should be cited as follows:**

Ramkumar, H., Ahmed, S.M., Syed, E., and Tuazon, J. (2009) Horseshoe kidney in an 80 year old with chronic kidney disease. *TheScientificWorldJOURNAL* **9**, 1346–1347. DOI 10.1100/tsw.2009.165.
